# In-Hospital Mortality and Costs of Added Morbidity in Heart Failure Patients at a University Hospital: A Retrospective Cross-Sectional Study

**DOI:** 10.3390/jcdd12050185

**Published:** 2025-05-15

**Authors:** Lourdes Raya Ortega, Jesús Martínez Tapias, María José Ferreras Fernández, Manuel Jiménez-Navarro, Almudena Ortega-Gómez, Miguel Romero-Cuevas, Juan José Gómez-Doblas

**Affiliations:** 1 Hospital Universitario Virgen de la Victoria, 29010 Málaga, Spain; lourdes.raya.ortega.sspa@juntadeandalucia.es; 2 Servicio de Admisión y Documentación Clínica, Hospital Universitario Virgen de las Nieves, 18014 Granada, Spain; 3 Servicio de Admisión y Documentación Clínica, Hospital Universitario Virgen de la Victoria, 29010 Málaga, Spain; 4 Unidad de Cardiología y Cirugía Cardiovascular, Hospital Universitario Virgen de la Victoria, 29010 Málaga, Spain; 5 Centro de Investigación Biomédica en Red de Enfermedades Cardiovasculares (CIBERCV-ISCIII), Instituto de Salud Carlos III, 28029 Madrid, Spain; 6 Instituto de Investigación Biomédica de Málaga y Plataforma en Nanomedicina (IBIMA Plataforma BIONAND), 29590 Málaga, Spain; 7 Departamento de Medicina y Dermatología, Universidad de Málaga, 20971 Málaga, Spain; 8 Servicio de Endocrinología y Nutrición, Hospital Universitario Virgen de la Victoria, 29010 Málaga, Spain; 9 Centro de Investigación Biomédica en Red de Fisiopatología de la Obesidad y Nutrición (CIBEROBN-ISCIII), Instituto de Salud Carlos III, 28029 Madrid, Spain

**Keywords:** heart failure, hospital mortality, comorbidity, angiotensin-converting enzyme inhibitors, angiotensin II receptor blockers, hospital costs

## Abstract

Background: Heart failure (HF) is a leading cause of hospital admissions and in-hospital mortality among the elderly. This study aims to characterize HF patients admitted to Virgen de la Victoria University Hospital (HUVV), identify factors associated with in-hospital mortality and analyze the impact of added morbidity on healthcare costs. Methods: A cross-sectional study was conducted using data from the Minimum Basic Data Set (MBDS) at HUVV. We included all discharges with a primary diagnosis of HF in 2021. Logistic regression analysis was employed to identify factors associated with mortality, and cost analysis was performed to assess the economic impact of added morbidity. Results: A total of 731 hospital discharges for HF were analyzed, with a mortality rate of 14.77%. Mortality was significantly associated with age ≥ 75 years (OR = 4.12; *p* < 0.001), high or extreme severity (OR = 2.26 and 8.10, respectively; *p* < 0.001), and more than 10 diagnoses at discharge (OR = 2.95; *p* < 0.01). Treatment with angiotensin-converting enzyme inhibitors (ACEIs) or angiotensin II receptor blockers (ARBs) was associated with a reduced risk of death (OR = 0.29; *p* < 0.001). Hospital-acquired morbidity occurred in 27.22% of patients, resulting in an additional cost of EUR 152,780.61, representing a 3.8% increase over the total hospitalization costs. Conclusions: In-hospital mortality in HF patients at HUVV is strongly associated with advanced age, disease severity, and multiple comorbidities. Treatment with ACEIs or ARBs was associated with a lower likelihood of in-hospital mortality. Preventable added morbidity was associated with increased healthcare costs, highlighting the importance of infection control measures and multidisciplinary management to potentially improve outcomes and reduce costs.

## 1. Introduction

Heart failure (HF) is the leading cause of hospital admission among individuals over 65 years old in Western countries, accounting for approximately 3–5% of hospital admissions in Spain [1]. This condition is associated with prolonged hospital stays and an increasing frequency of hospital readmissions [2]. The advanced age of these patients, along with the presence of comorbidities, contributes to longer hospitalization periods. Additionally, there is an increase in the rates of readmission, ranging between 29% and 59% within the first six months after hospital discharge. These factors, combined with a decline in quality of life, result in a significant cost burden on the healthcare system [3].

In terms of healthcare costs, HF demands a substantial number of resources, primarily related to treatment and hospitalization [4]. These costs are further exacerbated by episodes of additional morbidity during the hospital stay, particularly those arising from preventable factors, such as healthcare-associated infections (HAIs), which prolong hospital stays and increase mortality rates [5,6]. However, specific evidence on the additional costs generated by complications acquired during hospitalization is limited, largely due to challenges in analyzing the profile of frail patients with multiple comorbidities [7]. In the Spanish context, HF accounts for approximately 2.6% of hospital expenses and 3.8% of total healthcare spending, considering both outpatient follow-up and care [8].

HF is also one of the leading causes of mortality, although a decline in standardized mortality rates has been observed over time, partly attributable to improvements in available treatments [9]. Over the past two decades, this reduction has been significant, with a 50% decrease in mortality rates. For example, in Andalusia, HF mortality rates have decreased from 82 deaths per 100,000 inhabitants in 2000 to 42 deaths per 100,000 inhabitants in 2020. However, significant geographical variations exist, with Andalusia having the highest rate, followed only by the autonomous cities of Ceuta and Melilla. Moreover, this variability in mortality rates is observed at the provincial level and by gender, with Málaga being one of the provinces with the highest number of HF-related deaths, of which 61% correspond to women.

Despite advances in treatments, hospital mortality rates have shown little variation in recent years, remaining around 10%, primarily due to the advanced age and comorbidities of hospitalized patients [10]. Factors such as advanced age, the presence of multiple comorbidities, and the severity of the disease upon admission have been associated with poorer prognosis and higher mortality rates in HF patients [11,12]. Regarding pharmacological therapy, beta-blockers and angiotensin-converting enzyme inhibitors (ACEIs) have been shown to effectively reduce mortality and hospitalizations in patients with chronic HF [10]. The 2021 European Society of Cardiology (ESC) Clinical Practice Guidelines recommend ACEIs as first-line treatment to improve morbidity and mortality outcomes in patients with HF and reduced ejection fraction, while beta-blockers are considered essential to improving the prognosis of these patients [13].

Studies on quality indicators that assess the effectiveness of strategies to improve healthcare tend to focus more on processes than on patient outcomes [14]. However, a useful outcome indicator for monitoring the evolution of HF care is the in-hospital mortality rate [15].

To examine mortality in hospitalized patients, there is the Agency for Healthcare Research and Quality (AHRQ). This is a U.S. federal agency under the Department of Health and Human Services that develops and promotes evidence-based tools and indicators to improve the quality, safety, efficiency, and effectiveness of healthcare. Among its initiatives, it has established a set of Inpatient Quality Indicators (IQIs) derived from hospital administrative data, including the HF-related in-hospital mortality indicator (IQI 16), which is endorsed by the National Quality Forum (NQF) as a standardized measure of hospital performance. The AHRQ has developed quality indicators using data from hospitals’ Minimum Basic Data Set (MBDS) [16], defining 25 hospital admission quality indicators, known as Inpatient Quality Indicators (IQIs). IQI 16 establishes the in-hospital mortality rate for HF as the number of in-hospital deaths per 1000 discharges with HF as the primary diagnosis in patients over 18 years old. This indicator, endorsed by the National Quality Forum (NQF) as a top-tier metric, provides a valuable tool for hospital comparisons.

Since 2007, the Andalusian Health Service (SAS) has published AHRQ indicator data for its hospitals, including a comparison by hospital type. Additionally, the Hospital Program Contract includes reducing the HF mortality rate (IQI 16) as one of its annual objectives, evaluating compliance through the Standardized Ratio (RE) [17].

At the Virgen de la Victoria University Hospital (HUVV), in the last five years, the observed mortality rates for HF have been higher than expected. An improvement from 2020 to 2021 was noted, with a percentage reduction of 3.39% (from 18.18% to 14.79%), but it remains above the expected rate. In this context, the analysis of these data is a valuable tool for identifying factors associated with in-hospital HF mortality and improving the quality of care. In this context, the analysis of these data becomes a valuable tool for identifying factors associated with in-hospital HF mortality and improving the quality of care [18].

HF is currently the leading cause of hospitalization in Spain, especially among elderly patients, and it is associated with prolonged hospital stays, frequent readmissions, and substantial healthcare costs. This condition imposes a significant burden on healthcare systems, affecting both clinical care delivery and hospital resource management. We hypothesize that comorbidities play a critical role in driving in-hospital mortality and costs among patients with HF. By gaining deeper insight into these associations, hospital procedures and care pathways could be optimized to improve clinical outcomes and resource use efficiency.

The aim of this study is to characterize hospitalized HF patients using variables from the MBDS over a one-year period at the Virgen de la Victoria University Hospital (HUVV), identifying factors associated with mortality during hospital admission compared to those who were discharged. Additionally, this study aims to analyze preventable morbidity conditions acquired during hospitalization, estimating the added cost in comparison to the total cost of HF care from an economic perspective.

The results of this analysis can identify areas for improvement in the quality of care and patient safety, contributing to the reduction in morbidity and mortality in HF.

## 2. Methods

*Design:* A cross-sectional study based on data from the Minimum Basic Data Set (MBDS). In Spain, the Minimum Basic Data Set (MBDS), known as the Conjunto Mínimo Básico de Datos (CMBD), is a standardized data set approved in 1987 by the Interterritorial Council of the National Health System. It includes structured information collected at hospital discharge, such as demographic characteristics, diagnoses, procedures, and length of stay, codified using ICD-9-CM. It is widely used for epidemiological surveillance, healthcare planning, and hospital management.

*Study Subjects:* The unit of analysis was the hospital discharge due to HF. Information from all hospital discharges with a primary diagnosis of HF in patients aged 18 years or older from the MBDS of the Virgen de la Victoria University Hospital (HUVV) between 1 January and 31 December 2021 was selected.

*Dependent Variable:* The primary outcome was hospital discharge status (death or survival (another reason)).

*Independent Variables:* The independent variables included age, gender, health district, health center, country of birth, area of origin, circumstances of hospital admission or contact, the responsible service for the admission or contact, intensive care unit (ICU) admission, days of ICU stay, circumstances of hospital discharge, responsible discharge service, primary diagnosis, secondary diagnoses (up to 20), condition at the time of admission or at the onset of contact (POA) for diagnoses 1 to 20, external causes for the primary diagnosis (up to 5), surgical/diagnostic/therapeutic procedures (up to 20), pharmacological treatment, level of severity, level of mortality and cost of added morbidity.

For the analysis of the cost of added morbidity, the change in the severity level of the Diagnosis-Related Group (DRG) from admission to discharge was examined. This change in severity level identified the comorbidity acquired by the patient during the hospital stay. Each discharge was associated with a single DRG, and two additional classification criteria were introduced via the APR-DRG (All Patients Refined—DRG):

- Severity level: Four levels: mild, moderate, high, and extreme, based on the patient’s characteristics, the secondary diagnoses of the episode, and the procedures performed.
- Risk of mortality: Four levels: mild, moderate, high, and extreme, based on algorithms that combine the previously mentioned characteristics.

*Information Sources:* The Clinical Documentation Service of the HUVV. The criteria and methodology for calculating the IQI 16 indicator of the AHRQ were followed using the Enara Global Manager® software, version 1.1.8. application (Decisys, APR version v36.0). Data from the HUVV Pharmacy Service were used. The study database was cross-referenced with the pharmacological prescription database from the Receta XXI system. Average costs per DRG were calculated according to the APR-DRG version 32.0, based on the severity level established in the latest update of Order SCB/1421/2018 [19], of December 27, which modifies Annexes I, II, and III of Royal Decree 1207/2006 [20], of October 20, regulating the management of the Healthcare Cohesion Fund.

An evaluation of the diagnostic classification at admission and the appropriateness of the admitting service was performed, particularly in units other than Cardiology and Internal Medicine, as these are the usual services for admitting patients with this pathology.

*Statistical Analysis:* Univariate analysis: Quantitative variables were summarized using numerical summaries (mean, standard deviation, and interquartile range), while qualitative variables were presented in frequency tables. 

Bivariate analysis: Contingency tables were used to relate the independent variables to the dependent variable using the chi-squared test or Fisher’s exact test. A multivariate logistic regression analysis adjusted by severity level and mortality level was conducted to identify factors associated with in-hospital mortality in HF patients. Variable selection for the model was based on clinical and statistical significance, including those demonstrating a significant association in the bivariate analysis (*p* < 0.05). The overall model performance was assessed to ensure its adequacy in explaining the variability of the outcome variable. Potential interaction effects between variables were examined. Prior to inclusion in this study, a comprehensive data curation process was conducted to ensure completeness of essential data required for analysis. Patients with missing data in any of the essential variables were excluded from this study. When feasible, missing values were retrieved from alternative sources such as the electronic health record. Therefore, no statistical imputation techniques were applied, and only patients with a complete minimum data set were included in the final analysis.

*Ethical Considerations:* This study was approved by the Provincial Research Ethics Committee of Málaga (Reference: CEIM/2022/402) on 16 December 2022. All data were fully anonymized before analysis and handled in compliance with the General Data Protection Regulation (GDPR) and Spanish data protection laws.

## 3. Results

A thorough data cleaning process was conducted to evaluate the adequacy of admission, diagnosis, and service records. Factors associated with morbidity and mortality in patients with HF at the Virgen de la Victoria University Hospital (HUVV) were analyzed.

The descriptive variables of the sample are shown in Table 1. Univariate analysis revealed an almost equal gender distribution, with 49.93% male and 50.07% female patients. The variable “age” had a mean value of 76.8 years, though it was analyzed dichotomously, showing that 65.66% of patients were 75 years or older, of which 57.9% were women. The majority of patients were of Spanish nationality (89.47%), with the most frequent foreign nationality being British (2.46%).

The average hospital stay was 11.13 days, and 5.6% of patients were admitted to the Intensive Care Unit (ICU) during their hospitalization. A total of 731 hospital discharges due to HF were identified in 2021, with a mortality rate of 14.77%, and 85.23% of discharges were home-based.

Table 2 provides descriptive data based on the service of admission and discharge, severity and mortality levels, treatment, and the number of diagnoses. The Internal Medicine service handled the largest volume of admissions and discharges, with high levels of severity and mortality.

The multivariate logistic regression model demonstrated an adequate fit according to the Hosmer–Lemeshow test (*p* < 0.05). No significant multicollinearity was identified among the variables included in the logistic regression model. All variance inflation factors (VIF) were below 10 (range: 1.03–7.38), and the adjusted values normalized by the degrees of freedom dfVIF ranged from 1.016 to 1.648, well below the commonly accepted threshold of 2. The area under the receiver operating characteristic curve (AUC-ROC) was 0.804, indicating good discriminatory power of the logistic regression model.

A significant association was found between the risk of death and the independent variables, including age, service of admission and discharge, number of diagnoses, and pharmacological treatment, which are detailed below.

The logistic regression analysis showed that patients aged 75 years or older had a significantly higher risk of death (OR = 4.12; *p* < 0.001), as did those with extreme (OR = 8.10) or high (OR = 2.26) severity levels, compared to those with mild to moderate severity.

Although the variable “more than 10 diagnoses” was significantly associated with in-hospital mortality in the bivariate analysis (OR = 2.95; *p* < 0.01), it was no longer statistically significant in the multivariate model (OR = 1.30; *p* = 0.49). This attenuation suggests that the association was confounded by other variables included in the model, particularly severity and mortality level. These two indicators reflect the clinical complexity and prognosis of the patient and were included as adjustment factors. Once these variables were accounted for, the number of diagnoses no longer independently predicted mortality, indicating that it may act as a proxy for patient severity rather than an independent risk factor.

The relationship between the service of admission and discharge and mortality was notable. Treatment with ACE inhibitors (ACEI) or angiotensin II receptor blockers (ARB) was associated with a reduced risk of death (OR = 0.29; *p* < 0.001).

Table 3 summarizes the bivariate analysis of the independent variables in relation to in-hospital mortality.

In the multivariate logistic regression analysis, treatment with angiotensin-converting enzyme inhibitors (ACEIs) or angiotensin II receptor blockers (ARBs) was associated with a lower likelihood of in-hospital mortality (OR = 0.28; 95% CI: 0.17–0.46; *p* < 0.001). Similarly, patients aged ≥75 years had higher odds of mortality (OR = 2.59; 95% CI: 1.35–5.28; *p* < 0.01), and those discharged from the Internal Medicine department had significantly increased odds of in-hospital mortality compared to those discharged from Cardiology (OR = 8.78; 95% CI: 1.92–44.69; *p* < 0.01). Having more than ten diagnoses at discharge was not significantly associated with increased mortality risk (OR = 1.30; 95% CI: 0.63–2.93; *p* = 0.49). All ORs are presented with 95% confidence intervals to indicate statistical precision. The high odds ratio (OR = 15.60) for the high mortality level category underscores the strong association between clinical severity and in-hospital mortality in HF patients. This finding is consistent with clinical expectations, as the mortality level classification integrates multiple critical factors, including hemodynamic instability, the presence of multi-organ dysfunction, and the burden of comorbidities. All reported odds ratios are adjusted estimates obtained from multivariate models that included severity level and mortality level as covariates.

Patients categorized as high or extreme mortality level are often those with advanced-stage HF, severe decompensation, or systemic complications, which significantly increase their risk of death compared to patients with mild or moderate severity. The magnitude of this association underlines the importance of early identification and close monitoring of these high-risk patients, as well as the need for targeted therapeutic strategies to improve prognosis and reduce hospital mortality (Table 4).

During the hospital stay, 27.22% of patients with HF developed at least one additional condition (added morbidity, Table 5) that was not present upon admission. These conditions were identified using the Present On Admission (POA) indicator and typically included preventable issues, such as healthcare-associated infections. A total of 405 new diagnoses were recorded, affecting 199 patients, with an average of 2.04 additional diagnoses per patient. This added morbidity significantly impacted both patient outcomes and hospital costs.

The most common added morbidities were urinary tract infections (UTIs), which accounted for 5.68% of all added diagnoses and affected 3.15% of all HF hospitalizations. Other frequent morbidities included phlebitis and thrombophlebitis, which represented 2.96% of added diagnoses and acute renal failure, affecting 1.50% of patients. Infections associated with medical devices, such as cardiac implants, also contributed to the added morbidity burden, appearing in 1.09% of cases.

These additional complications not only increased the length of hospital stay but also imposed a considerable financial burden on the healthcare system. The economic cost of hospital-acquired morbidity was estimated by calculating the change in the severity level of the Diagnosis-Related Group (DRG) from admission to discharge. Each patient was assigned a single DRG and severity level at both time points. The unit costs per DRG and severity level were obtained from the Spanish Ministry of Health’s official reimbursement pricing, as established in Order SCB/1421/2018 [19]. This regulation defines reference prices for hospital services by DRG category and level of severity for the national healthcare system.

The total cost at admission for all HF discharges was EUR 4,020,610.49. After accounting for changes in DRG severity due to added morbidity, the total cost at discharge increased to EUR 4,173,391.10, representing an additional EUR 152,780.61 (3.8%). The average cost per patient at admission was EUR 5501.52 and increased to EUR 5709.15 at discharge.

## 4. Discussion

This study focuses on identifying the characteristics and factors associated with in-hospital mortality in patients with HF at the Virgen de la Victoria University Hospital (HUVV). The results provide valuable information about the complexity of care for these patients and highlight key areas for improvement to improve clinical outcomes and reduce mortality.

Consistent with the existing literature, our findings confirm that HF predominantly affects elderly patients, with more than 65% of cases occurring in individuals over 75 years of age [21,22]. Additionally, this group carries a substantially higher risk of mortality compared to younger patients, with the risk up to four times greater. Although there was a slight increase in mortality among women over 75 years, adjusted analyses revealed that men had a higher risk of death, although this difference did not reach statistical significance.

A notable finding from our study is that most patients presented a high burden of comorbidities, with up to 80% of them having more than 10 diagnoses at discharge (Table 3). This multimorbidity was significantly associated with an increased risk of mortality, underscoring the importance of addressing not only HF but also concomitant medical conditions in the management of these patients. Additionally, a significant proportion of patients did not receive pharmacological treatment with ACE inhibitors (ACEIs) or angiotensin II receptor blockers (ARBs), despite clinical practice guidelines recommending their use in HF patients to improve survival [23,24].

It is important to acknowledge certain limitations of this study, particularly regarding the availability of clinical data, the quality of medical records, and the study design. While the data set provided valuable insights into in-hospital mortality and healthcare costs in HF patients, some discrepancies in admission records were identified during data cleaning. Specifically, 21.2% of cases showed an inadequacy in the recorded reason for admission, and 19.1% in the admission service, suggesting a degree of diagnostic misclassification. However, after a thorough coding revision, the HF mortality rate was adjusted to 15.1%, ensuring greater accuracy in the analysis.

Additionally, although this study includes key variables related to patient demographics, comorbidities, and severity at admission, we acknowledge that the inclusion of additional clinical parameters could further enrich the analysis and provide an even more precise assessment of disease severity. Nevertheless, the results remain consistent and aligned with the existing literature [25], reinforcing their validity despite these limitations. Future research incorporating these clinical variables could help refine risk stratification and enhance our understanding of factors influencing in-hospital outcomes in HF patients.

Another limitation is the single-center and retrospective design of this study, which may affect the generalizability of the findings to other settings. However, it is important to highlight that this study was conducted at Hospital Universitario Virgen de la Victoria (HUVV), the reference hospital for the province of Málaga, Spain, which provides specialized care to a broad and diverse population. As a tertiary referral center, the HUVV receives and manages a high volume of complex cases, making the study findings particularly relevant for hospitals with similar healthcare structures and patient profiles. Despite this, differences in healthcare infrastructure, admission policies, and treatment protocols in other regions or hospital settings may influence patient outcomes, which should be considered when extrapolating these results.

The distribution of hospital discharges by service revealed a significant involvement of Internal Medicine in the management of HF patients, compared to Cardiology and Cardiovascular Surgery. This difference may reflect insufficient interdisciplinary coordination in HF management at our center, suggesting the need to improve collaboration between units to ensure comprehensive and high-quality care for these patients. Additionally, given the complexity of HF and its management, especially in stressful contexts such as the COVID-19 pandemic, it is crucial to review and optimize admission and discharge protocols from emergency departments, as well as improve continuity of care between the different specialties involved in the care of these patients.

Specialized HF units play a fundamental role in the comprehensive care of these patients, offering a range of services from in-hospital consultations to outpatient follow-up. However, it is essential that all HF patients receive an adequate level of care, regardless of the admitting unit or the responsible service. Ensuring that all patients benefit from the available resources and advances in HF management is critical to improving clinical outcomes and quality of life in these patients [26].

An important aspect highlighted by this study is the significant incidence of added morbidity during the hospitalization of patients with HF. More than 27% of patients developed at least one new condition during their hospital stay, with urinary tract infections (UTIs), phlebitis, and acute renal failure as the most common. These conditions, categorized as preventable hospital-acquired morbidities, not only complicate the clinical management of HF patients but also extend hospital stays and increase the risk of mortality. As described in previous research, the development of hospital-acquired infections, particularly in elderly and frail patients, is often associated with the prolonged use of invasive devices and a lack of adequate infection control measures [26]. These diagnoses can significantly impact clinical outcomes and healthcare costs, highlighting the importance of implementing effective infection prevention and control strategies in hospital settings.

The economic implications of added morbidity are substantial. In our study, approximately 3.8% of the total cost of HF care was attributable to the management of these additional conditions, underscoring the financial burden on the healthcare system. The average cost per patient with added morbidity was significantly higher compared to those without complications, reflecting the additional resources required for extended hospital stays, specialized care, and treatment of infections and other complications. This highlights the urgent need for the implementation of robust infection prevention strategies, which have the potential not only to improve clinical outcomes but also to significantly reduce healthcare costs [27,28].

The economic burden of HF, as highlighted in our study, is consistent with findings from recent research in Spain, which underscores the high costs associated with HF hospitalizations, followed by indirect costs and outpatient care. A study analyzing healthcare resource utilization and costs in newly diagnosed HF patients in Spain showed that hospitalizations account for the majority of HF-related costs, with a 50% reduction in these costs over a four-year follow-up period, attributed to improved management and survival bias. However, the study also concluded that there is still significant room for improvement in the use of guideline-recommended therapies, which could further reduce hospitalizations and healthcare costs associated with HF.

Investing in patient safety programs and preventive measures could lead to a substantial reduction in avoidable hospital costs [29]. Preventable conditions, such as catheter-associated UTIs and hospital-acquired phlebitis, represent lapses in high-quality care delivery and significantly contribute to non-quality-related expenses. By addressing these preventable issues, healthcare systems can both improve the quality of care provided to HF patients and decrease the financial strain associated with prolonged hospital stays and the treatment of hospital-acquired complications. Additionally, the economic burden of managing these complications reinforces the importance of early detection and management of added morbidity, which could result in improved patient outcomes and lower overall costs [30].

## 5. Conclusions

This study underscores the multifactorial nature of in-hospital mortality and costs of heart failure (HF) patients admitted to a tertiary hospital. Advanced age, clinical severity, and the presence of multiple comorbidities emerged as key factors associated with worse outcomes. Furthermore, the inadequate use of evidence-based pharmacological treatments, particularly ACE inhibitors and ARBs, was linked to higher mortality risk, highlighting opportunities for therapeutic optimization. The identification of a high rate of added morbidity—largely preventable conditions such as infections—further stresses the importance of improving hospital practices, especially in infection control and interdisciplinary coordination.

From an economic perspective, added morbidity contributed significantly to the overall cost of care, revealing a substantial margin for improving efficiency in hospital resource utilization. Ensuring accurate admission documentation and promoting adherence to guideline-recommended therapies are essential steps to reduce variability in clinical practice and enhance patient safety.

Altogether, these findings point to the need for integrated clinical pathways, standardized risk stratification protocols, and investment in preventive strategies. This work provides an evidence base to inform quality improvement programs, guide clinical decision making, and support the development of healthcare policies aimed at reducing the burden of HF and improving patient outcomes.

## Figures and Tables

**Table 1:** Descriptive variables of the study sample.

Variable	Number	Percentage (%)	95% CI
Sex			
Male	365	49.93	46.31–53.56
Female	366	50.07	46.44–53.69
Age			
<75 years	251	34.34	62.22–69.11
≥75 years	480	65.66	30.89–37.78
Country of Origin			
Spain	654	89.47	87.24–91.69
Ohers	77	10.53	8.31–12.76
United Kingdom and North Ireland	18	2.46	
Morocco	12	1.64	
Other (21 countries)	47	6.43	

**Table 2:** Descriptive variables by service (admission and discharge), severity, mortality levels, and treatment.

Variable	Number	Percentage (%)	95% CI
Admission Service			
Internal Medicine	354	48.43	44.80–52.05
Cardiology and Cardiovascular Surgery	242	33.11	29.69–36.52
Other Units	135	18.47	15.65–21.28
Discharge Service			
Internal Medicine	400	54.72	51.11–58.33
Cardiology and Cardiovascular Surgery	276	37.76	34.24–41.27
Other Units	55	7.52	5.61–9.44
Severity Level			
Extreme	125	17.10	14.37–19.83
High	293	40.08	36.53–43.63
Mild-Moderate	313	42.81	39.23–46.41
Mortality Level			
Extreme	105	14.36	11.82–16.91
High	263	35.98	32.50–39.46
Mild-Moderate	363	49.66	46.03–53.28
Treatment with ACEI or ARB			
Yes	518	70.86	67.57–74.16
No	213	29.14	25.84–32.43
Treatment with Beta-blockers			
Yes	495	67.71	64.33–71.10
No	236	32.29	28.90–35.67
Number of Diagnoses at Discharge > 10			
Yes	577	78.93	75.98–81.89
No	154	21.06	18.11–24.02

**Table 3:** Analysis of independent variables by mortality at hospital discharge.

Variable	Death	Other: Home Discharge. Voluntary Discharge or Escape	OR	*p*
Age				
≥75 years	94 (19.6%)	386 (80.4%)	4.12	<0.001
<75 years	14 (5.6%)	237 (94.4%)	1	
Sex				
Male	49 (13.4%)	316 (86.6%)	0.81	0.304
Female	59 (16.1%)	307 (83.9%)	1	
Severity Level				
Extreme	46 (36.8%)	79 (63.2%)	8.10	<0.001
High	41 (14.0%)	252 (86%)	2.26	
Mild-Moderate	21 (6.7%)	292 (93.3%)	1.00	
Mortality Level				
Extreme	45 (42.9%)	287 (82.5%)	4.22	<0.001
High	46 (17.5%)	60 (57.1%)	15.60	
Mild-Moderate	17 (4.7%)	346 (95.3%)	1.00	
Admission Service				
Cardiology and Cardiovascular Surgery	17 (7.06%)	225 (93.0%)	0.71	<0.001
Internal Medicine	78 (22.0%)	276 (78.0%)	2.65	
Other Units	13 (9.6%)	122 (90.4%)	1.00	
Discharge Service				
Cardiology and Cardiovascular Surgery	12 (43%)	264 (95.7%)	0.16	<0.001
Internal Medicine	84 (21%)	316 (79.0%)	0.95	
Other Units	12 (21%)	43 (78.2%)	1.00	
Treatment (ACEI/ARB)				
Yes	50 (9.7%)	468 (90.3%)	0.29	<0.001
No	58 (27.2%)	155 (72.8%)	1	
More than 10 diagnoses				
Yes	98 (16.9%)	479 (83.1%)	2.95	<0.01
No	10 (6.5%)	144 (93.5%)	1	

**Table 4:** Logistic regression analysis of independent factors associated with hospital mortality. Multivariate logistic regression models were used to estimate adjusted odds ratios (aORs), including severity level and mortality level as covariates.

Variable	OR	95% CI	*p*
≥75 years	2.59	1.35–5.28	<0.01
Male Sex	1.18	0.72–1.94	0.5
More Than 10 Diagnoses	1.30	0.63–2.93	0.49
Treatment (ACEI/ARB)	0.28	0.17–0.46	<0.001
Admission Service			
Cardiology and Cardiovascular Surgery	Ref.		
Internal Medicine	0.26	0.05–1.18	0.08
Other Units	0.11	0.02–0.47	<0.01
Discharge Service			
Cardiology and Cardiovascular Surgery	Ref.		
Internal Medicine	8.78	1.92–44.69	<0.01
Other Units	12.73	3.26–56.73	<0.001

**Table 5:** Most frequent added morbidities not present on admission.

Diagnosis	Number	% of Total Diagnoses	% of Total Episodes
Urinary tract infection	23	5.68%	3.15%
Phlebitis and thrombophlebitis	12	2.96%	1.64%
Acute renal failure; other types	11	2.72%	1.50%
Infection from cardiac/vascular devices	8	1.98%	1.09%
Muscle atrophy; multiple locations	8	1.98%	1.09%
Pneumonitis due to inhalation of food/vomit	7	1.73%	0.96%
Macroscopic hematuria	7	1.73%	0.96%
Klebsiella pneumoniae infection	6	1.48%	0.82%
Total	405	100%	

## Data Availability

The datasets used and/or analyzed during the current study are available from the corresponding author upon reasonable request.
